# The Moroccan human mutation database

**DOI:** 10.4103/0971-6866.45004

**Published:** 2008

**Authors:** Ilham Ratbi, Alae-eddine Gati, Abdelaziz Sefiani

**Affiliations:** Department of Medical Genetics, National Institute of Health, 27 avenue Ibn Batouta, 9654 Rabat, Morocco

Sir,

The mutation spectrum observed for any gene or disorder often varies between population groups. The settling of Morocco was marked by a great diversity. The main ethnic groups are the Berbers and Arabs, but there have also been currents of Phoenicians, Romans, Vandals, Byzantines, Moriscos, sub-Saharian Africans, and European remnants of the colonial period[1]. In addition, there is a high degree of consanguinity (up to 25% of consanguineous marriage and 50% between first-degree cousins)[2]. In 2007, the Moroccan population was estimated over 33 million, according to the last census of the Moroccan authorities. Morocco is an emigration country: nearly 1.8 million Moroccans live abroad, in particular in France, Italy, and Belgium. Information on inherited disorders in Morocco is not extensive. For a long time, infectious and environmental diseases were the highest priority for the Moroccan Health System. This policy does explain the reason why molecular studies on genetic diseases among the Moroccan population are rare. There are only few detailed studies on the prevalence of some diseases such as familial Mediterranean fever[3], cystic fibrosis[4], betathalassemia[5]. But a huge amount of sporadic data is available for more than 100 genetic diseases and syndromes.

Because of its vocation, our Department of Medical Genetics has a special interest in genetic diseases among the Moroccan population. Thus, we managed to build up in 2007 the first Moroccan Human Mutations Database (MoHuMuDa), freely available at the address: http://www.sante.gov.ma/Departements/INH/MoHuMuDa/index.htm. It is devoted to the collection of reported human mutations in Mendelian diseases identified in the native Moroccan population, or in patients from Moroccan origin living abroad. Polymorphisms, but not microsatellite markers, which had been investigated in samples associated with specific phenotypes or diseases are also included. The resources of mutations are Pubmed (http://www.ncbi.nlm.nih.gov/pubmed/) and other online databases, scientific meetings, and unpublished data directly submitted to the database. MoHuMuDa is a user-friendly tool, hosted in the site of the National Institute of Health (Rabat, Morocco). On the Home Page, the users are informed on the latest changes, the number of entries from different countries in the database, and the number of genetic diseases and mutations listed. From the Home Page, they can access the different parts of the database by using shortcut links to the column “search” by disease index, using the first letter of the disease name. Diseases are categorized alphabetically (Online Mendelian Inheritance in Man OMIM names, accessible at http://www.ncbi.nlm.nih.gov/omim/), with all reported mutations for each gene stated in the same page. For each mutation disease, a specific table precises the name of the gene, the OMIM number of the disease, the published DNA, and amino-acid change, the proper nomenclature, the number of chromosomes, the frequency of the mutation, and the source of the data. The Home Page also gives access to the column “links” that lists numerous relevant addresses including the National Center for Biotechnology Information (NCBI), the Human Genome Organization, Genetic Alliance and links for some Moroccan structures and patients' associations. There is also a column for submission which can be used to send mutation data to the database curator. From the section “contact”, the users can communicate with the manager team of the database. Thanks to MoHuMuDa, scientists and other related professionals will have the opportunity to take into account mutation data on Moroccan subjects in their future research planning. They could also direct studies on molecular epidemiology, diagnose and prevent genetic disorders through simple and economical molecular biology techniques, in particular when these are recurrent genetic mutations among the Moroccan population.

## References

[1] Bosch E, Calafell F, Pérez-Lezaun A, Clarimón J, Comas D, Mateu E (2000). Genetic structure of north-west Africa revealed by STR analysis. Eur J Hum Genet.

[2] Attazagharti N, Hami H, Soulaymani A (2006). Consanguinité dans la région du Gharb au Maroc. Biologie et Santé.

[3] Belmahi L, Sefiani A, Fouveau C, Feingold J, Delpech M, Grateau G (2006). Prevalence and distribution of MEFV mutations among Arabs from the Maghreb patients suffering from familial Mediterranean fever. C R Biol.

[4] Ratbi I, Génin E, Legendre M, Le Floch A, Costa C, Cherkaoui-Deqqaqi S (2008). Cystic fibrosis carrier frequency and estimated prevalence of the disease in Morocco. J Cyst Fibros.

[5] Lemsaddek W, Picanco I, Seuaneus F, Nogueira P, Mahmal L, Benchekroun S (2004). The beta-thalassemia mutation/haplotype distribution in the moroccan population. Hemoglobin.

